# Cranial Ultrasound in the Management of Hydrocephalus in Newborns: A Case Series

**DOI:** 10.3390/children12040419

**Published:** 2025-03-27

**Authors:** Bogdan Florin Gonț, Loredana Mitran, Vlad Dima, Simona Vlădăreanu

**Affiliations:** 1 Faculty of Medicine, Carol Davila University of Medicine and Pharmacy, 050474 Bucharest, Romania; 2 Neonatology Clinic, Department of Obstetrics and Gynecology, Faculty of General Medicine, “Elias” Emergency University Hospital, 011461 Bucharest, Romania; 3 ENT Department, “Elias” Emergency and University Hospital, 011461 Bucharest, Romania; 4 Neonatology Department, Obstetrics and Gynecology, Filantropia Clinical Hospital, 011132 Bucharest, Romania

**Keywords:** hydrocephalus, ventriculomegaly, preterm neonate, term neonate, cranial ultrasound

## Abstract

Background: Hydrocephalus in preterm and term newborns is a condition with an important impact on medical care and the neurological development of patients, with high expenditures regarding daily care. Imaging nowadays provides valuable information regarding the aetiology of the condition, and it represents a great aid in monitoring the development of the patients. Materials and methods: In this article, we present the cases of five patients with hydrocephalus, for which different imaging methods were used to detect and treat the underlying aetiology, emphasizing the cranial ultrasound examination. Results: The results provided valuable information regarding the utility and feasibility of ultrasound. Moreover, Prechtl’s Assessment of General Movements is yet another useful tool, which in correlation with the use of cranial ultrasound can provide good insight regarding the evolution of the affected newborns. The Kurjak Antenatal Neurodevelopmental Test (KANET, KANE Test) comes in handy in the case of prenatal neurological assessments. Conclusions: Ultrasound examination proves to be a worthy tool capable of detecting the aetiology; however, in several cases, a complementary imaging examination might be needed. The therapeutic approach should take into consideration the diverse aetiology of the disease.

## 1. Introduction

Hydrocephalus is a clinical and neuroradiographic condition characterized by abnormal cerebrospinal fluid (CSF) accumulation, which may or may not alter the intracranial pressure. When the pressure remains normal or low, the compensatory mechanisms involve cortical tissue and cranial expansion. This condition significantly impacts medical care and neurocognitive development, with high morbidity and mortality rates [1,2,3]. Pindrik et al. suggested that “ventriculomegaly” be used strictly as a radiographic term [3]. Fetal ventriculomegaly is classified as mild (10–12 mm), moderate (13–15 mm), or severe (>15 mm) based on lateral ventricle measurements via ultrasound [4,5,6,7,8].

Hydrocephalus is classified by etiology as primary (syndromic or idiopathic) or secondary to another condition [1]. Primary hydrocephalus is linked to genetic factors affecting neurodevelopment and is associated with conditions such as neural tube defects, Dandy–Walker syndrome, Chiari malformation, and arachnoid cysts [1,9,10,11,12,13,14].

Secondary hydrocephalus results from infections, hemorrhage, or trauma. Post-hemorrhagic hydrocephalus (PHH) is the most common form in developed countries, often following intraventricular or subarachnoid hemorrhage, particularly in preterm infants under 37 weeks or with very low birth weight (<1500 g) [1,3,15,16]. In developing countries, neonatal infections are the leading cause [17,18,19]. Intrauterine infections (e.g., CMV, toxoplasmosis, enteroviruses) and medications such as misoprostol, metronidazole, antidepressants, and isotretinoin have also been implicated [6,20,21,22,23,24,25]. Other causes include congenital defects [17,18,19].

The clinical signs of increased intracranial pressure include rapid head circumference growth, bulging fontanelle, widened cranial sutures, upward gaze palsy, lethargy, vomiting, and episodic bradycardia [3,17,26]. The diagnosis and follow-up rely on ultrasound, MRI, and CT scans [3,26]. The treatment aims to address the underlying cause, with ventriculoperitoneal shunting, endoscopic third ventriculostomy (ETV), or choroid plexus cauterization (CPC) as standard interventions [3,26]. Neurological assessments, such as the general movement examination, help track preterm infants’ development, with specific movement patterns correlating to ventricular size and brainstem abnormalities [27].

## 2. Material and Methods

We present you five cases of newborns with hydrocephalus, four preterms and one at term. All cases studied presented different etiologies and different risk factors implied in developing the condition. Four of the cases were born at Medlife Life Memorial Hospital Bucharest, while one case was observed after one month of life. The period of the observance was between 2020–2023. Ultrasound examinations were performed with the help of Siemens X100 (Siemens Healthineers, Erlangen, Germany) and GE Healthcare Vivid S60 (General Electric HealthCare, Waukesha, West Milwaukee and Madison, WI, USA) instruments.

### 2.1. Case 1

Case 1 involved a preterm newborn at 31 weeks of gestation, delivered via emergency C-section due to fetal distress, who presented at birth in critical condition, with apnea, pallor, and bradycardia. Initial resuscitation was performed using a mask and bag ventilation (T-piece resuscitator), followed by endotracheal intubation and external cardiac massage.

**Figure 1:**
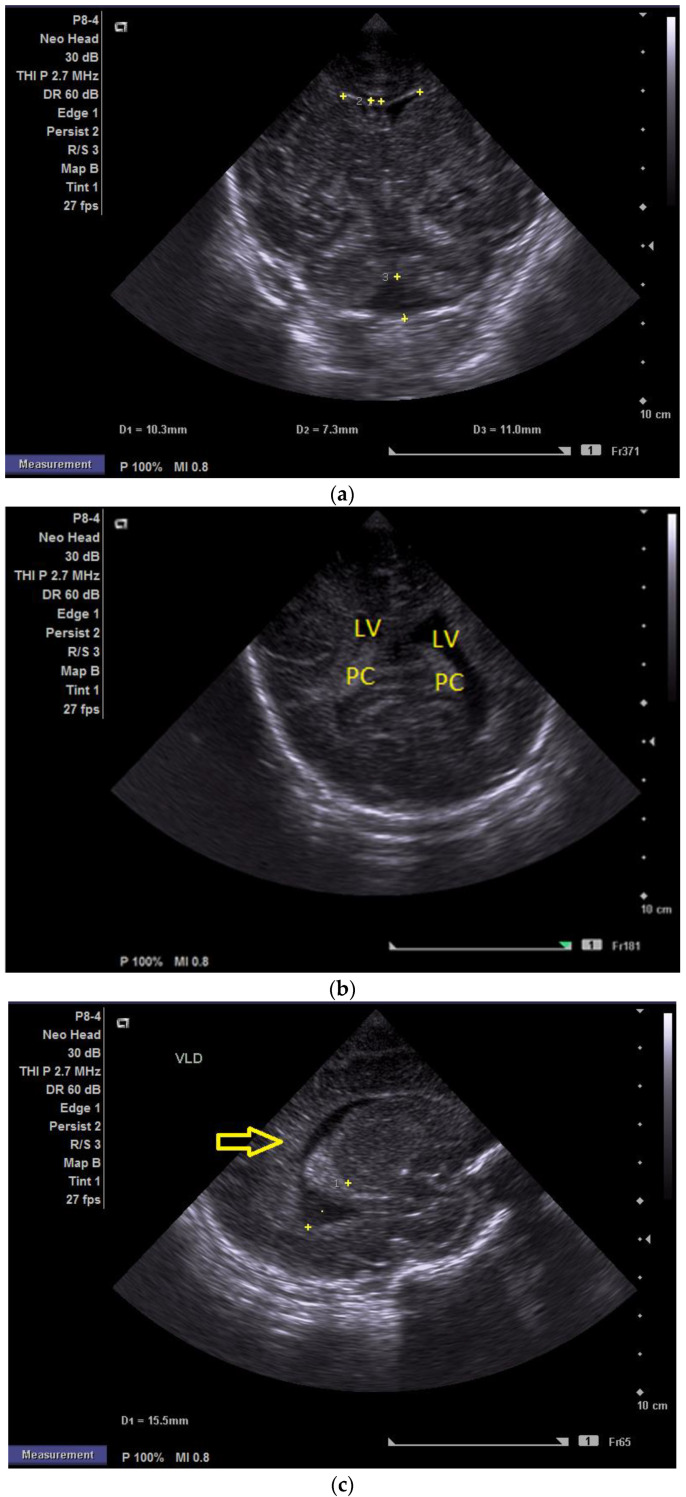

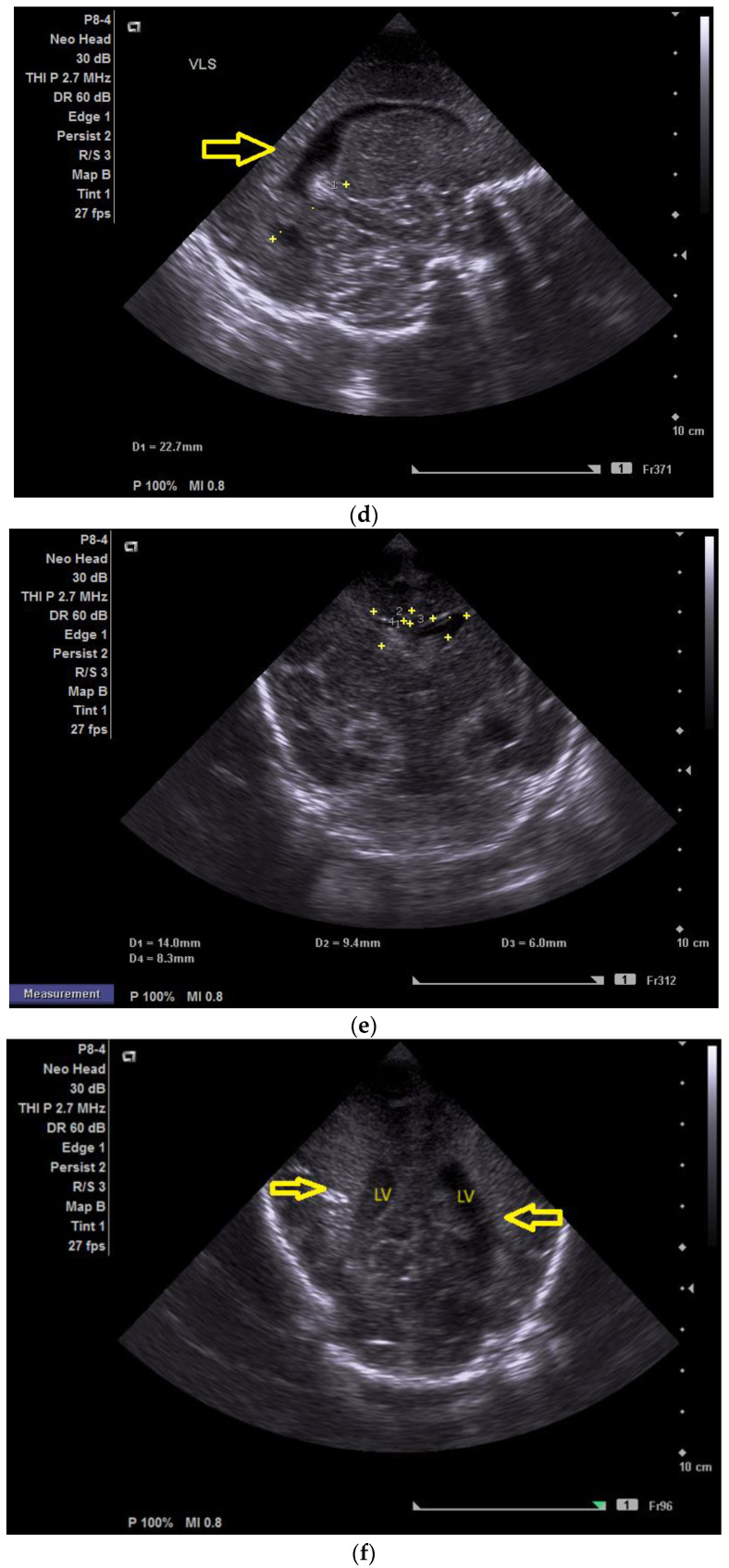

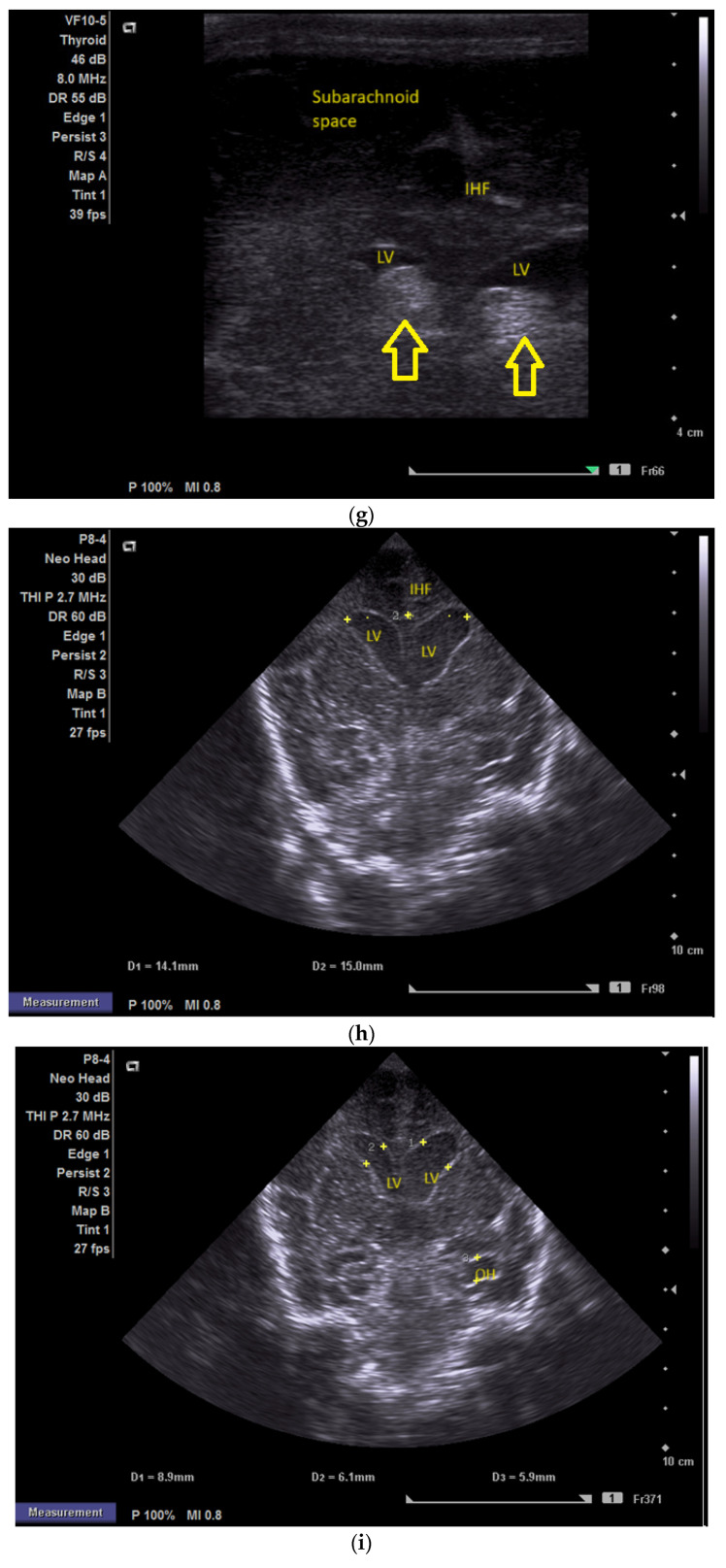

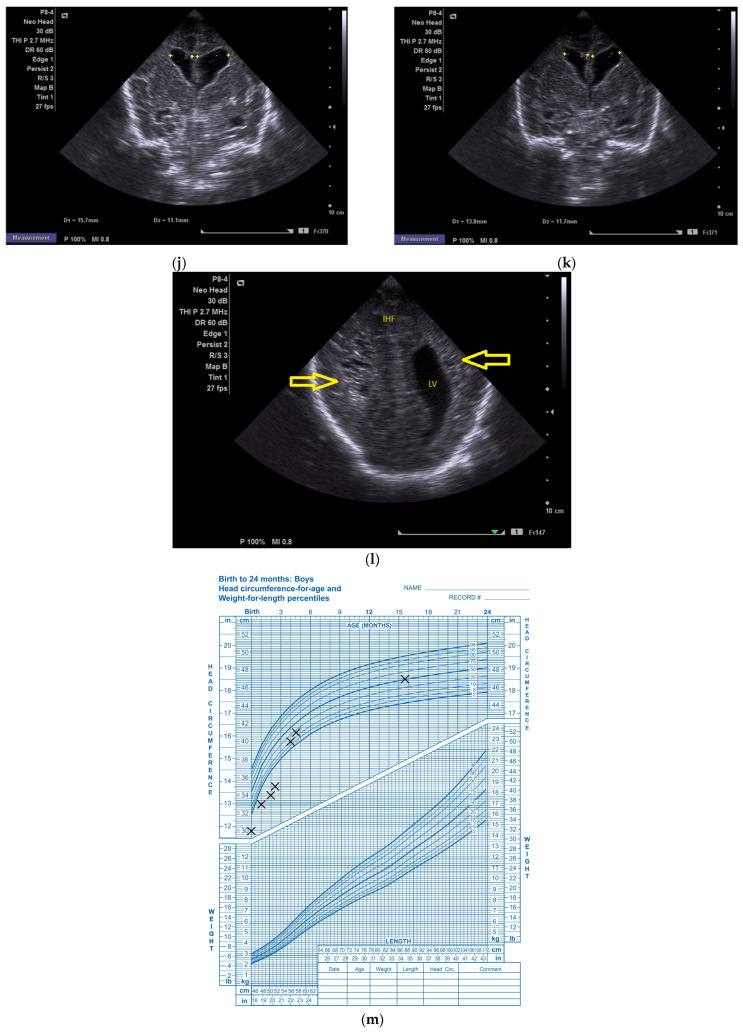
(**a**) Cranial ultrasound. Day one. Coronal view. D1 and D2—Levine index measurement. D3—cisterna magna measurement (personal image collection). (**b**) Cranial ultrasound. Day one. Coronal view. Lateral ventricle dilatation. LV—lateral ventricles. PC—plexus choroidus (personal image collection). (**c**) Cranial ultrasound. Day one. Right lateral ventricle. D1—thalamo-occipital distance measurement. Yellow arrow—parieto-occipital hyperechoic images (personal image collection). (**d**) Cranial ultrasound. Day one. Left lateral ventricle. D1—thalamo-occipital distance measurement. Yellow arrow—parieto-occipital hyperechoic images (personal image collection). (**e**) Day three. Cranial ultrasound. Coronal view. Ventricular asymmetry. D1 and D2—Levine index measurement. D3 and D4—anterior horn width measurement. Left ventricular enlargement (personal image collection). (**f**) Day three. Cranial ultrasound. Coronal view. Ventricular asymmetry (left ventricle larger than right ventricle). Yellow arrows—periventricular hyperechoic images are becoming more visible (personal image collection). (**g**) Day three. Cranial ultrasound. Coronal view. Linear probe. Ventricular asymmetry (left ventricle larger than right ventricle). LV—lateral ventricle (anterior horns). IHF—interhemispheric fissure. Yellow arrows—hyperechoic images in the thalamic-caudate groove (suggestive of a germinal matrix hemorrhage) (personal image collection). (**h**,**i**) Day 13. First LP. Cranial ultrasound. Coronal view. Up (before LP). D1 and D2—Levine index measurement. LV—lateral ventricles (anterior horns). IHF—interhemispheric fissure. Down (after LP). Dimensions of ventricles were reduced after LP. D1 and D2—anterior horn width. LV—lateral ventricles (anterior horns). OH—occipital horn (personal image collection). (**j**,**k**) Day 19. Second LP. Cranial ultrasound. Coronal view. Dimensions of ventricles were reduced after LP. Left (before LP). D1 and D2—Levine index measurement. Right (after LP). D1 and D2—Levine index measurement (personal image collection). (**l**) Day 39. Cranial ultrasound. Coronal view. Yellow arrows—periventricular cystic hyperechoic images—suggesting periventricular leukomalacia (cystic form) (personal image collection). (**m**) Head circumference-for-age. “X” symbols represent the measurements performed from birth and follow-up examinations. Initially, the head circumference was under the curve (personal image collection) [28].

At two minutes of life, with a heart rate of 120 bpm, chest compressions were discontinued, and the patient continued to be ventilated via endotracheal tube. The newborn was then transported while ventilated in an incubator to the neonatal intensive care unit (NICU), where SIMV ventilation was initiated, along with surfactant administration. The day one cranial ultrasound (CUS) showed bilateral periventricular hyperechoic images and enlargement of the lateral ventricles (Figure 1a–d).

After 72 h, the patient was extubated. The CUS showed bilateral IVH (grade III Volpe/Papile) (Figure 1e,f).

During the in-stay, two lumbar punctures (LP) were performed (Figure 1h–k).

In evolution, our patient presented bilateral ventricular dilatation and bilateral PVL (cystic form, grade III) (Figure 1l). The neurological assessment established eye contact and the infant could hold its head during the traction maneuver. The passive tone assessment showed the “scarf sign”, with the elbow at the midline bilaterally and a popliteal angle of 90°. The deep tendon reflexes (DTRs) were symmetrical.

Clinically, hypertonicity of the legs and axial muscles was present. At the one-year follow-up examination, the patient showed developmental delay and strabismus. At the three-year follow-up examination, cerebral palsy could be observed, which was bilateral and spastic with a predominance of the legs. The classification according to the Gross Motor Function Classification System (GMFCS) was grade III.

### 2.2. Case 2

Case 2 involved a 30-week preterm newborn from a mother with preeclampsia, delivered via C-section under general anesthesia (Apgar Score—6 at 1 min, 7 at 5 min), who required resuscitation at birth (T-piece resuscitator) due to apnea, generalized cyanosis, and hypotonia. After the initial resuscitation maneuvers, there was an improvement in color and tone but the newborn exhibited irregular breathing and respiratory distress, leading to intubation, respiratory support, and admission to the neonatal intensive care unit (NICU).

The infant was continuously monitored and received an endotracheal surfactant within the first hour of life, followed by nasal CPAP support. Due to progressively increasing oxygen requirements and worsening respiratory distress, mechanical ventilation was initiated, and a second dose of surfactant was administered at 15 h of life.

After extubation at five days of life, the newborn continued nasal CPAP support until day 7 and received oxygen therapy in an incubator until day 10.

**Figure 2:**
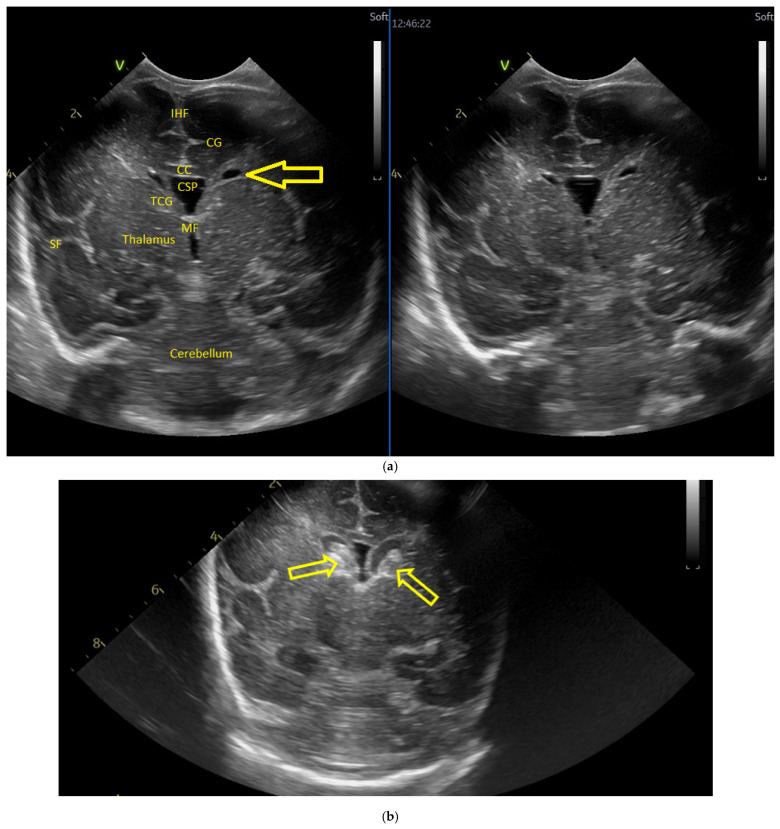

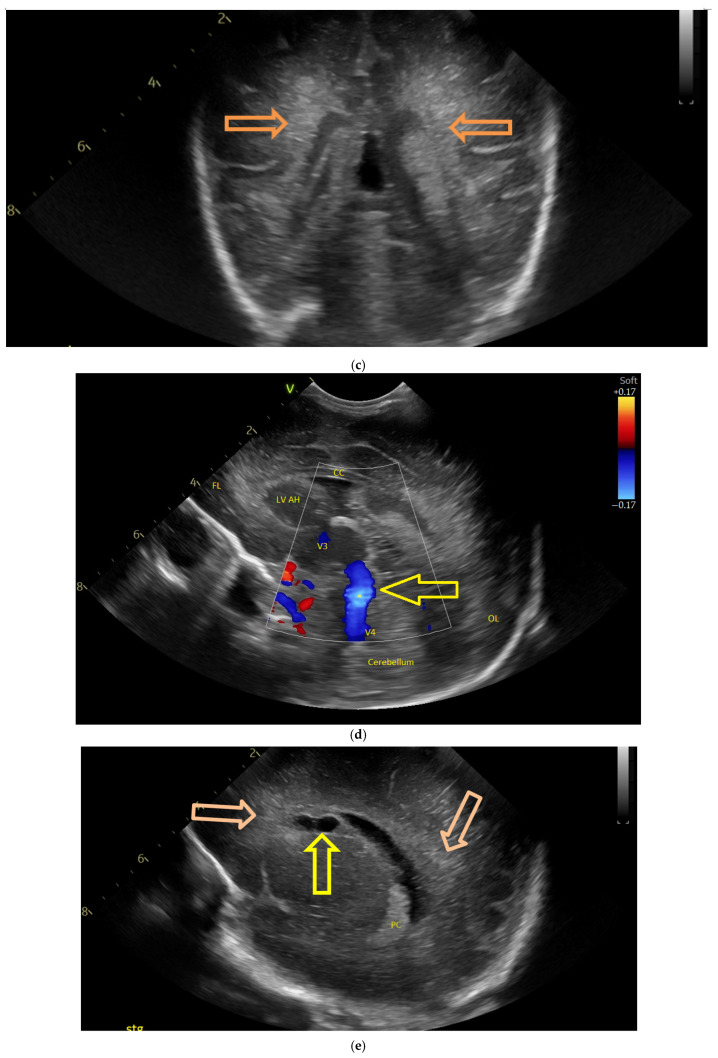

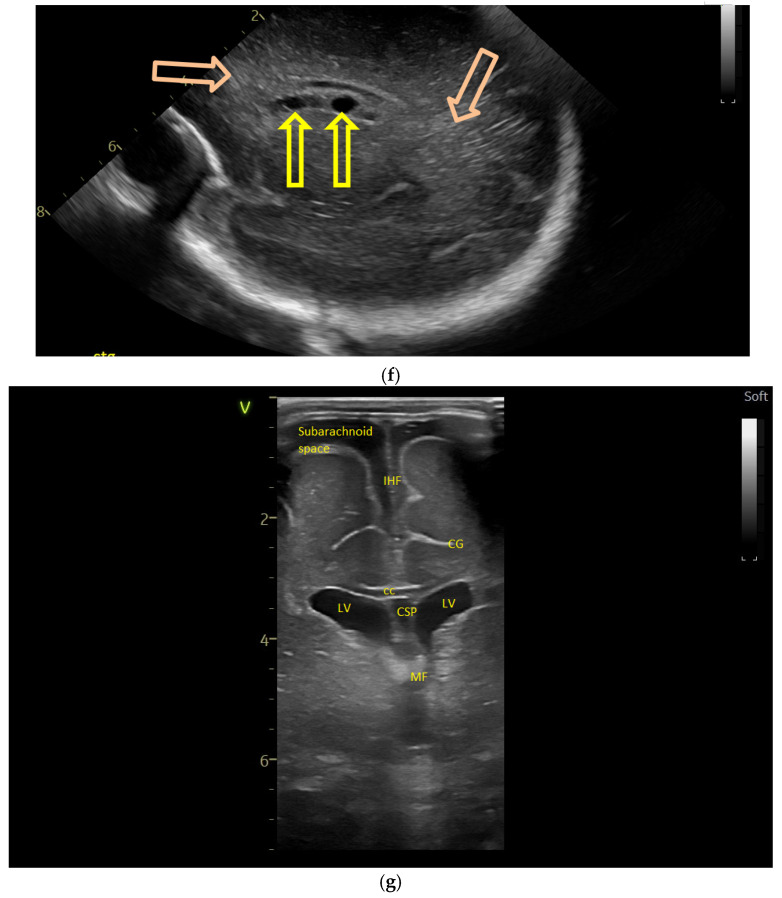

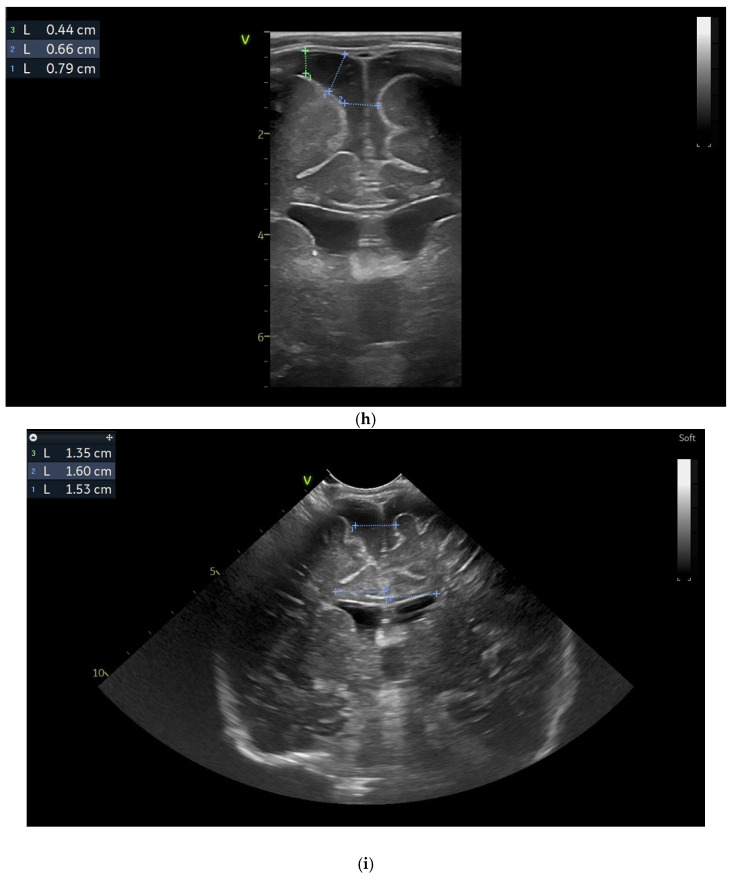

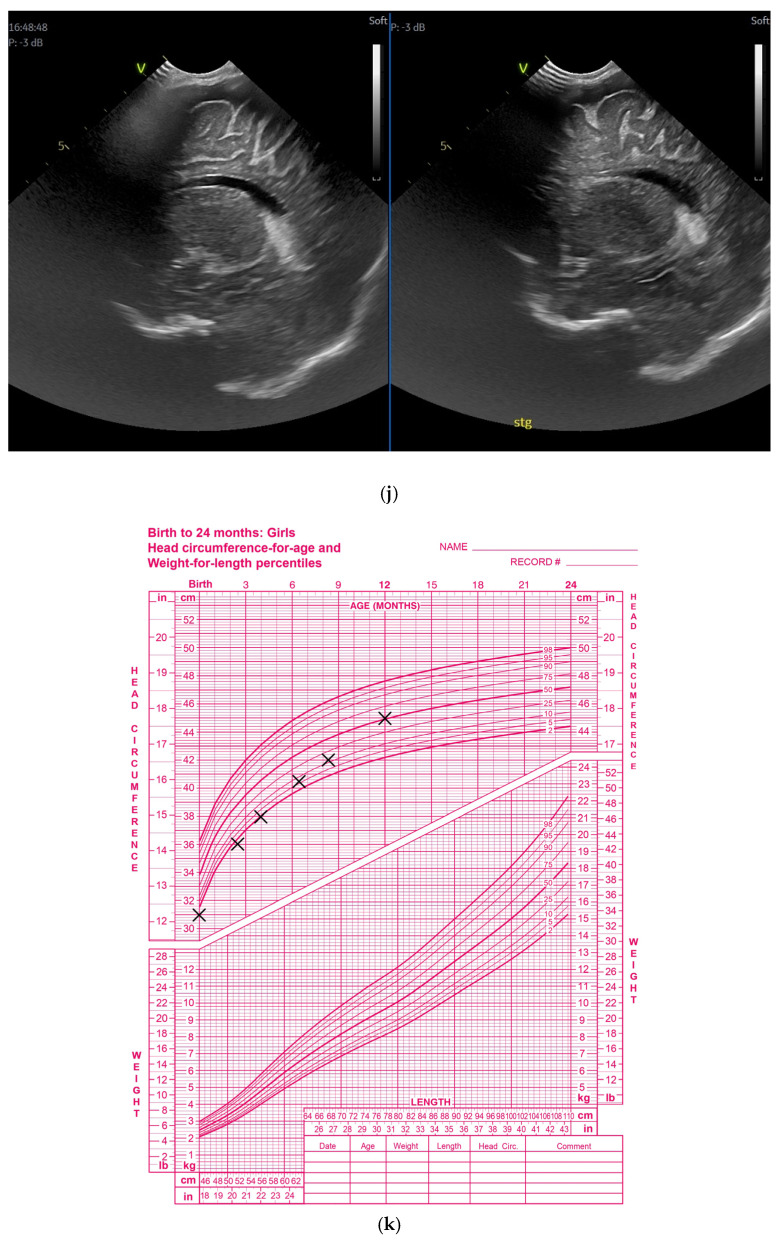
(**a**) Day one. Cranial ultrasound. Sagittal view. A left frontal conatal cyst can be observed. IHF—interhemispheric fissure. CG—cingulate gyrus. CC—corpus callosum. CSP—cavum septum pellucidum. TCG—thalamic-caudate groove. MF—Monroe foramina. SF—Sylvian fissure. Yellow arrow—conatal cyst (personal image collection). (**b**) Day three. Cranial ultrasound. Coronal view. Bilateral ventricular dilatation. Yellow arrows—hyperechoic images can be seen at the level of the thalamic-caudate groove and within the ventricles (personal image collection). (**c**) Day three. Cranial ultrasound. Coronal view. Orange arrows—bilateral periventricular hyperechoic images (personal image collection). (**d**) Day three. Cranial ultrasound—Doppler examination. Sagittal view. CC—corpus callosum. FL—frontal lobe. LV AH—lateral ventricle anterior horn. V 3—third ventricle. V 4—fourth ventricle. OL—occipital Lobe. Yellow arrow—presence of color Doppler signal within the Sylvian aqueduct (personal image collection). (**e**) Day 19. Cranial ultrasound. Sagittal view (left). Yellow arrows—conatal cysts. Pink arrows—PVL lesions (personal image collection). (**f**) Day 19. Cranial ultrasound. Sagittal view (left). Yellow arrows—conatal cysts. Pink arrows—PVL lesions (personal image collection). (**g**) Follow-up examination at term corrected age. Cranial ultrasound. Coronal view. Slight dilation of the lateral ventricles. IHF—interhemispheric fissure. CG—cingulate gyrus. CC—corpus callosum. CSP—cavum septum pellucidum. LV—lateral ventricle. MF—Monroe foramina (personal image collection). (**h**) Second follow-up examination. Cranial ultrasound. Coronal view. Dilation of interhemispheric fissure and subarachnoid space. D1—sinus–cortical width measurement. D2—interhemispheric width measurement. D3—cranio-cortical width measurement (personal image collection). (**i**) Third follow-up examination. Cranial ultrasound. Coronal view (left). D1—dilation of interhemispheric fissure. D2, D3—Levine index measurement (personal image collection). (**j**) Cranial ultrasound. Sagittal view (right). Normal brain structures (personal image collection). (**k**) Head circumference-for-age. “X” symbols represent the measurements performed from birth and follow-up examinations (personal image collection) [28].

The day one of life CUS revealed the presence of a left frontal conatal cyst with no other modification (Figure 2a).

On day three of life, the CUS displayed a bilateral germinal matrix hemorrhage with more than 50% of the ventricular volume being occupied by blood. Periventricular hyperechoic images could be observed as well (Figure 2b,c).

On the fourth day of life, a Doppler examination showed the presence of a signal within the third ventricle (Figure 2d).

A series of four lumbar punctures were performed during the patient’s in-stay to relieve the intracranial pressure—expressed as a bulging fontanelle. The first three lumbar punctures presented hemorrhagic CSF, while the last lumbar puncture showed sero-citrine CSF. Afterwards, the ventricular dimensions stabilized; however, small visible PVL lesions were visible on the CUS examination, in addition to the two conatal cysts that were observed (Figure 2e,f).

The first follow-up CUS examination was performed at the term-equivalent age (corrected gestational age) and revealed a slight dilation of the lateral ventricles (Figure 2g). The neurologic examination showed normal development for the corrected gestational age and mild plagiocephaly.

The next follow-up examination was performed two months from birth at the term-equivalent age and the CUS examination showed dilation of the interhemispheric fissure and the subarachnoid space (Figure 2h). Plagiocephaly, torticollis, mild motor development delay, and hypertonia in the lower limbs were observed.

Four months after the first follow-up examination, the CUS revealed normal brain structures. The general movement examination showed absent fidgety movements, while the neurological examination showed a global developmental delay with hypertonia of the limbs predominantly at the level of the left side of the body (Figure 2i,j).

On the fourth follow-up examination, the dilations were still visible. The neurological examination revealed mild trapezius muscle retraction and mild hypertonia at the level of the inferior limbs. The fifth follow-up examination—performed at one year after birth—revealed mild motor developmental delay.

### 2.3. Case 3

Case 3 involved a preterm newborn (33 3/7 weeks of gestation) with antenatal lateral ventricle asymmetry, for which a left ventricular hyperechoic image with the fetal MRI was performed at 28 weeks of gestation, born from a mother with recurrent UTIs (urinary tract infections) with E. Coli who was extracted through C-section and required resuscitation at birth (T-piece resuscitator). A nasal CPAP was provided for almost 36 h and antibiotic therapy was initiated (Vancomycin^®^ and Cefotaxime^®^ for three days).

**Figure 3:**
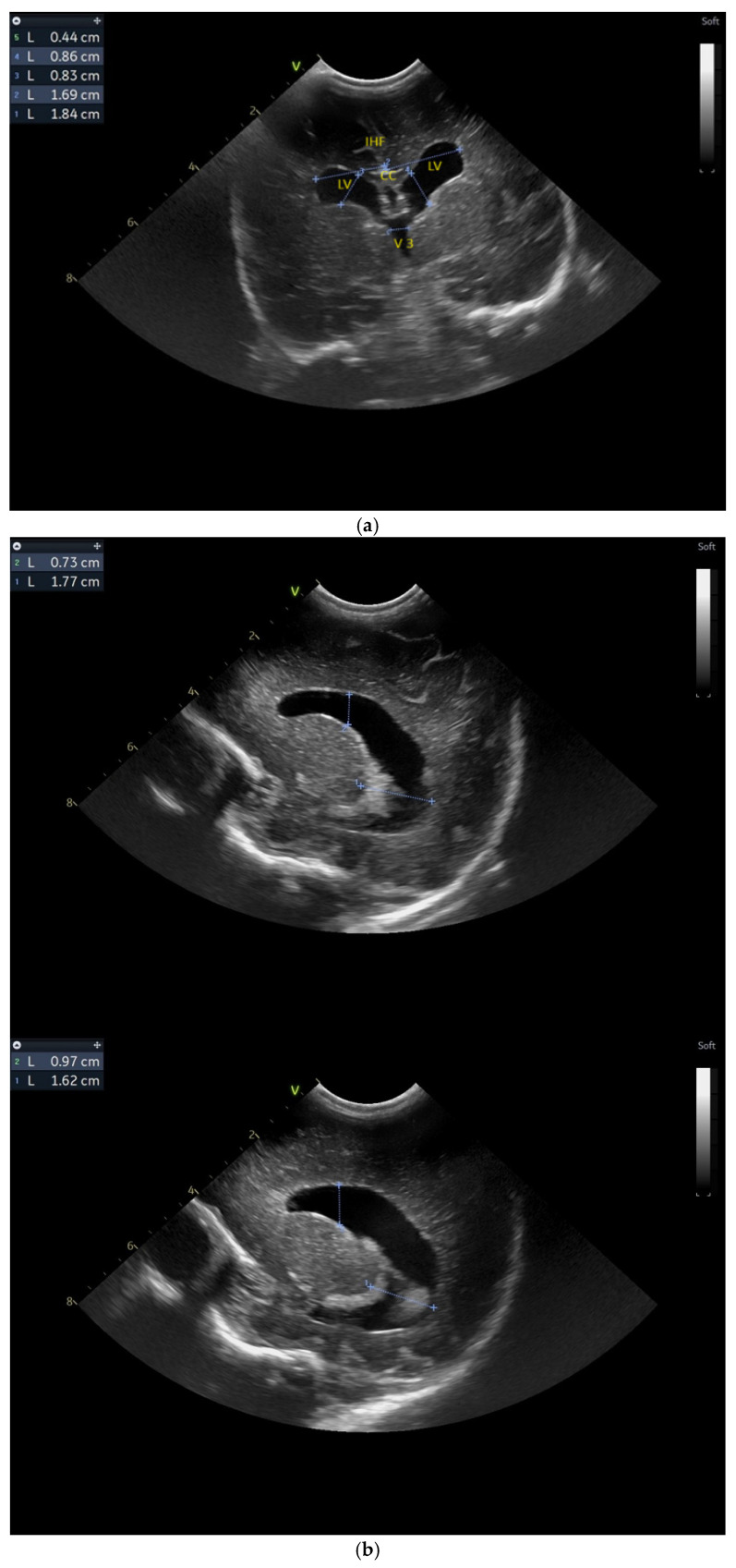

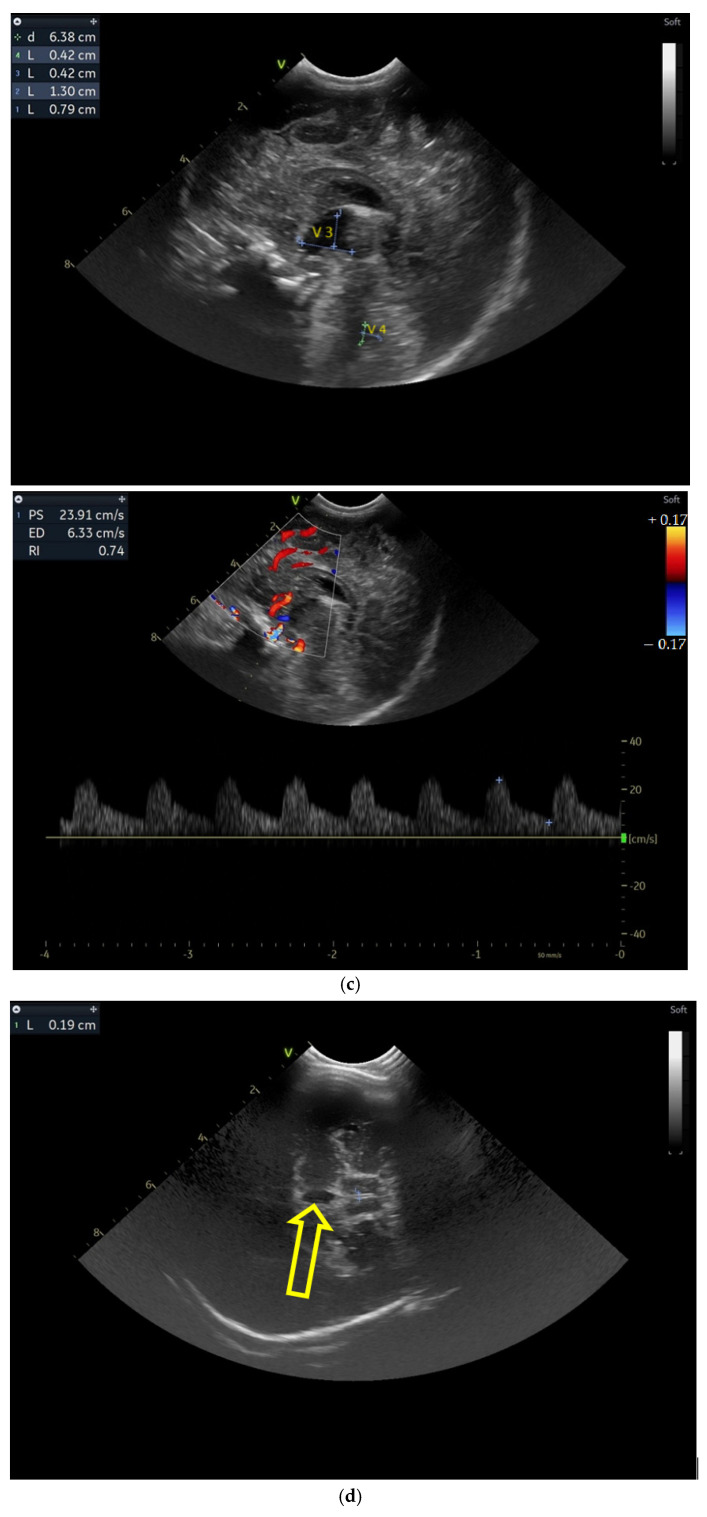

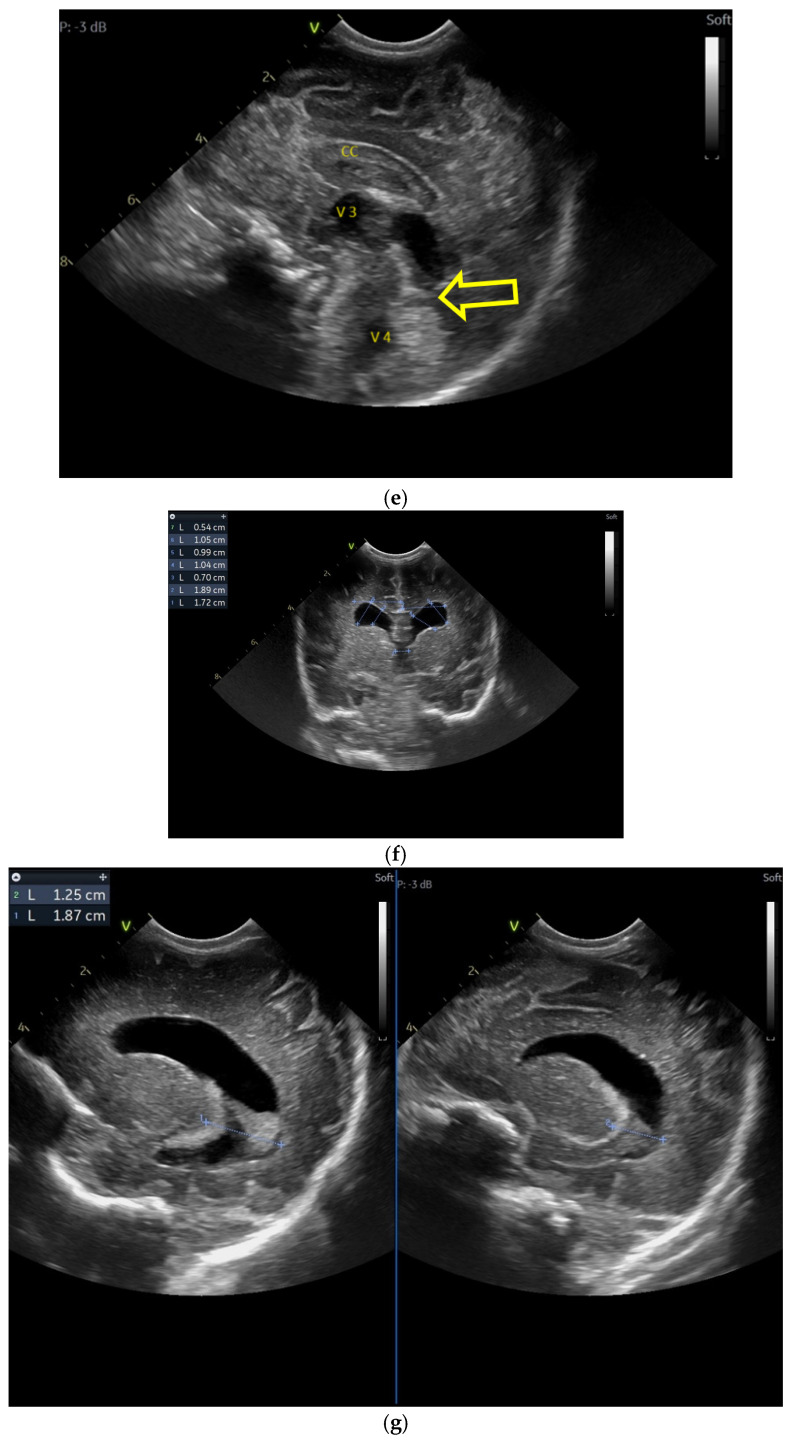

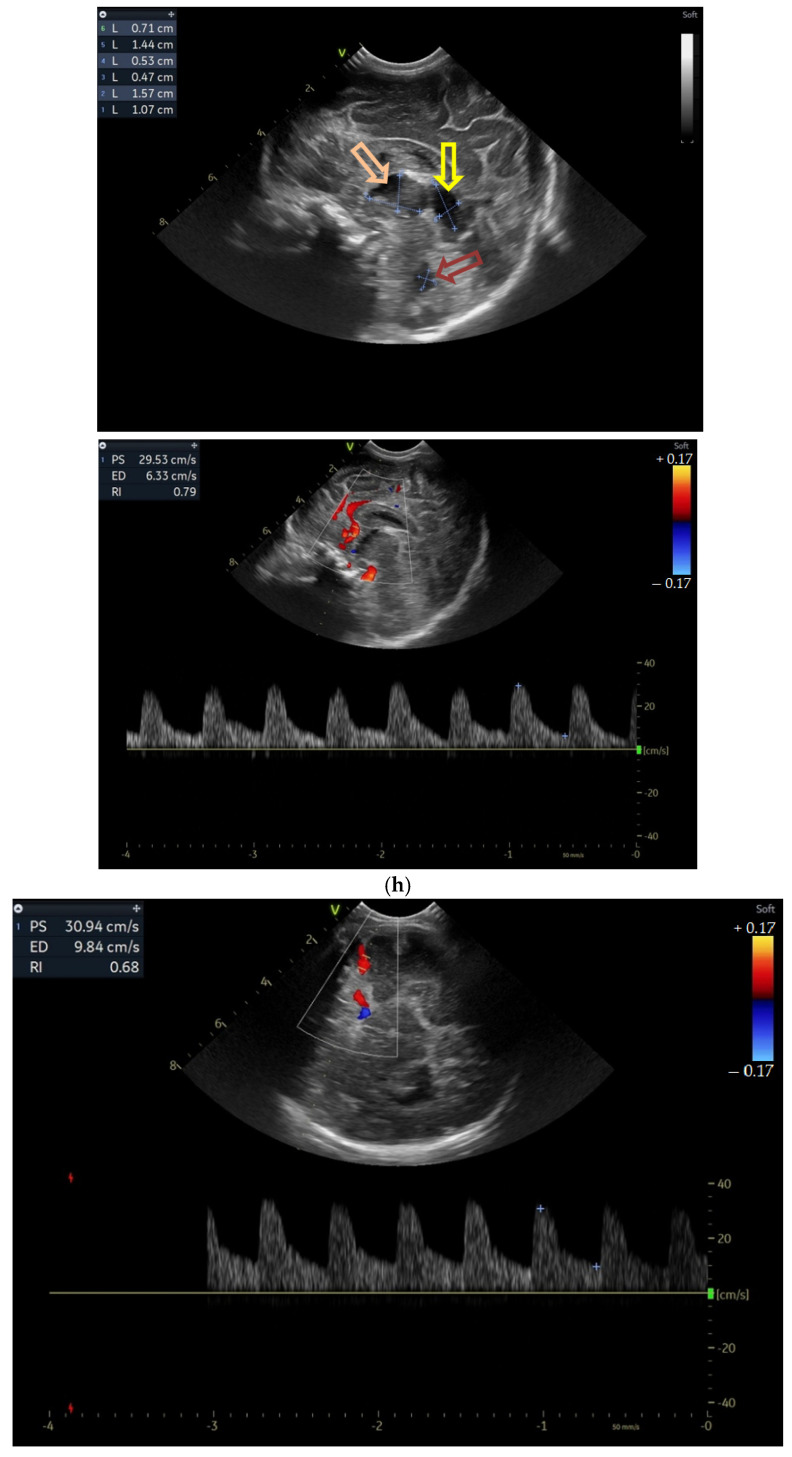

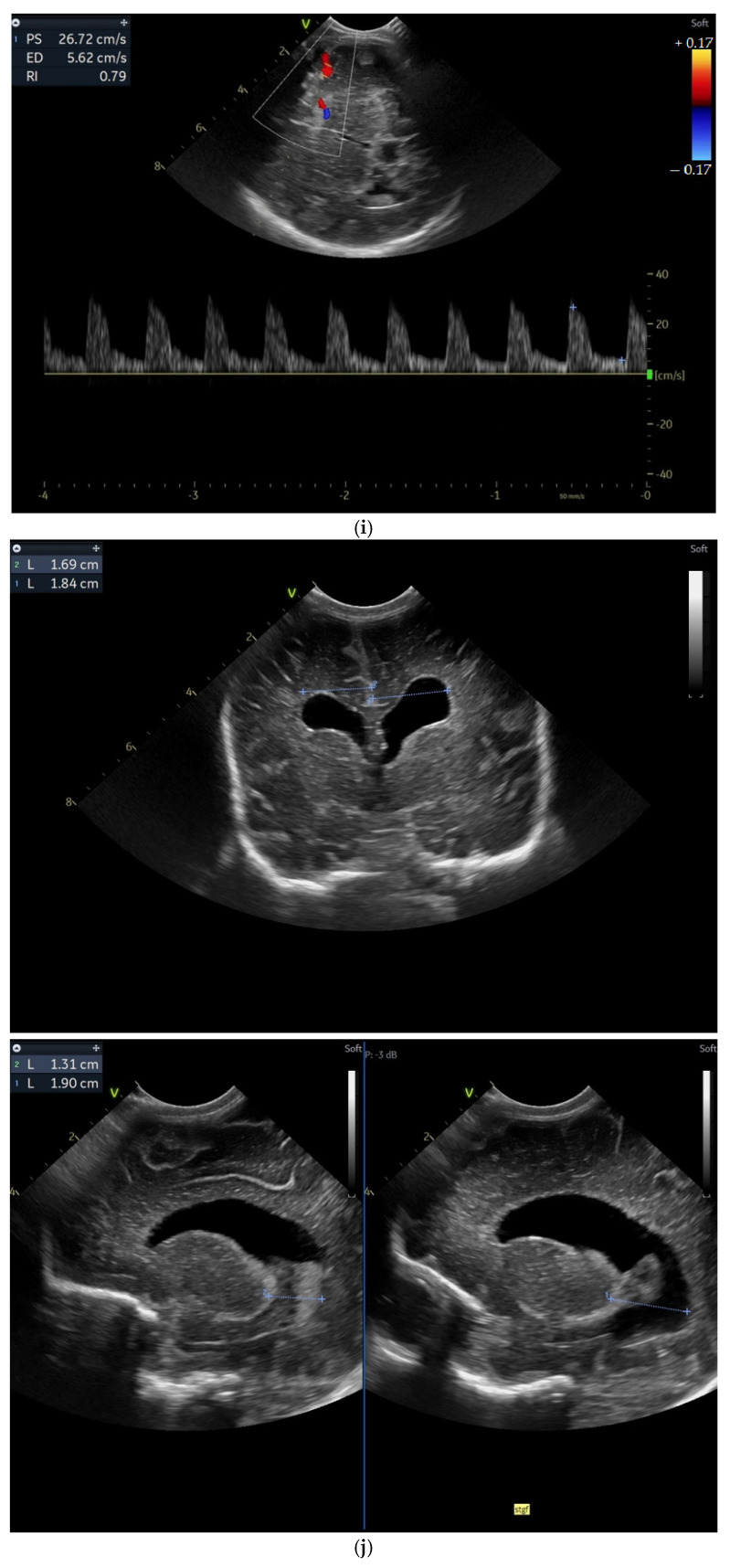

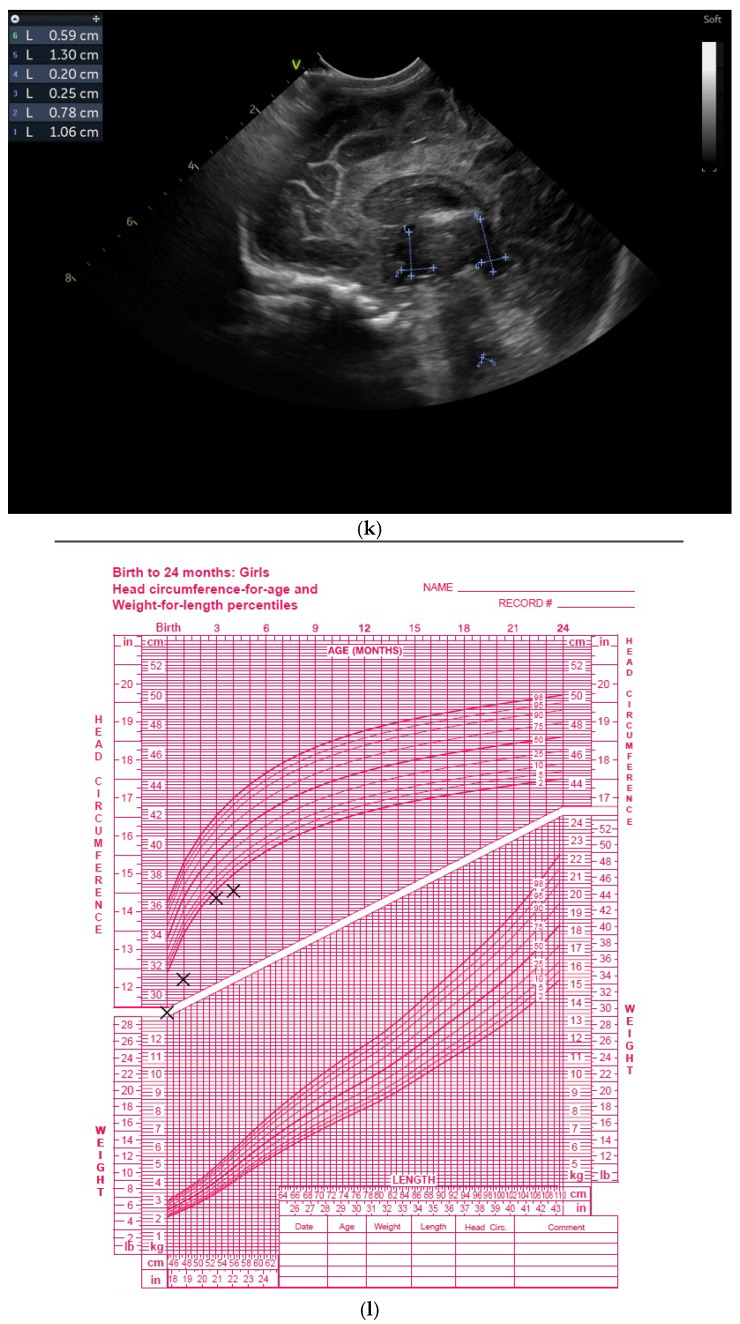
(**a**) Cranial ultrasound. Coronal view. Enlargement of the lateral ventricles with hyperechoic and third ventricle dilation. D1, D2—Levine index measurement. D3, D4—anterior horn width measurement. D5—third ventricle (personal image collection). (**b**) Cranial ultrasound. Sagittal view. Up—left ventricle. D1, D2—lateral ventricle measurements and thalamo-occipital horn width. Down—right ventricle. D1, D2—lateral ventricle measurements and thalamo-occipital horn width (personal image collection). (**c**) Cranial ultrasound. Sagittal view. Up—enlargement of the third and fourth ventricles. Measurements of the ventricles. Down—measurement of the resistive index (RI) performed on the anterior cerebral artery (ACA) using Doppler flow measurements. RI—0.74 (personal image collection). (**d**) Cranial ultrasound. Transtemporal view. Yellow arrow—enlargement of the Sylvian aqueduct (personal image collection). (**e**) Cranial ultrasound. Sagittal view. Yellow arrow—arachnoid cyst posterior to the third ventricle (personal image collection). (**f**) Cranial ultrasound. Coronal view. Lateral ventricles and third ventricle enlargement. D1, D2—Levine index measurement. D3, D6—right lateral ventricle measurement (anterior horn width). D4, D5—left lateral ventricle measurement (anterior horn width) (personal image collection). (**g**) Cranial ultrasound. Sagittal view. Left—left ventricle. D1—thalamo-occipital distance measurement. Right—right ventricle. D2—thalamo-occipital distance measurement (personal image collection). (**h**) Cranial ultrasound. Coronal view. Up—orange arrow—third ventricle. Red arrow—fourth ventricles. Yellow arrow—arachnoid cyst. Down—resistive index measured on the anterior cerebral artery. RI—0.79 (personal image collection). (**i**) Cranial ultrasound. Transversal view. Up—RI precompression value on the middle cerebral artery performed with Doppler examination. RI—68. Down—RI post-compression value on the middle cerebral artery performed with Doppler examination. RI—0.79 (personal image collection). (**j**) Cranial ultrasound. Up—coronal view. L1, L2—Levine index measurement of the lateral ventricles. Down—sagittal view. L1, L2—measurement of the thalamo-occipital distance (personal image collection). (**k**) Cranial ultrasound. Sagittal view. L1, L2—measurement of the third ventricle. L3, L4—measurement of the fourth ventricle. L5, L6—measurement of the arachnoid cyst (personal image collection). (**l**). Head circumference-for-age. “X” symbols represent the measurements performed from birth and follow-up examinations. Head circumference was still under the curve during follow-up examinations (personal image collection) [28].

The first CUS was performed 24 h after birth and showed dilation of the third and fourth lateral ventricles, enlargement of the Sylvius aqueduct, and the presence of an arachnoid cyst (Figure 3a–e).

The CUS examination performed at 48 h after birth showed increases in the diameters of all ventricles. The resistive index presented an increase as well. The bregmatic fontanelle started bulging. The dimensions of the Sylvian aqueduct were not modified (Figure 3f–h).

The CUS examinations performed at four days and seven days after birth showed stabilization regarding the values of the diameters. The pre- and post-compression values of the resistive index of the middle cerebral artery showed a delta-RI of 0.11 (pre—0.68, post—0.79, Figure 3i).

After discharge, at three weeks after birth (follow-up examination), the ventricle measurements showed wider ventricles and the arachnoid cyst was still present (Figure 3j,k).

The next follow-up examination performed a week later showed a delta-RI of 0.12 and an increase in the cranial perimeter (+ one cm since the last follow-up).

At 12 weeks of life (five week corrected age), ventricle dilations were still present. Neurological examinations showed axial and limb hypotonia. The general movement examination showed a poor repertoire with a General Movement Optimality Score (GMOS) of 19. At 18 weeks of life (11 week corrected age), the neurological exam revealed that the infant presented plagiocephaly and could keep eye contact, and during the traction-to-sit maneuver showed hypotonia of the neck muscles. In the prone position, the infant was unable to hold the head up. The passive tone assessment showed the “scarf sign”, with the elbow at the midline bilaterally and a popliteal angle of 90° on the right and 110° on the left. Deep tendon reflexes were present. The general movement (GM) assessment revealed absent fidgety movements, with a Motor Optimality Score (MOS) of 13.

### 2.4. Case 4

Case 4 involved a 37-week gestational age infant born from a mother with Toxoplasma gondii infection during pregnancy who was delivered through C-section due to failed spontaneous birth. The IgM antibodies were negative at birth and the newborn was treated for Toxoplasma one month after birth.

**Figure 4:**
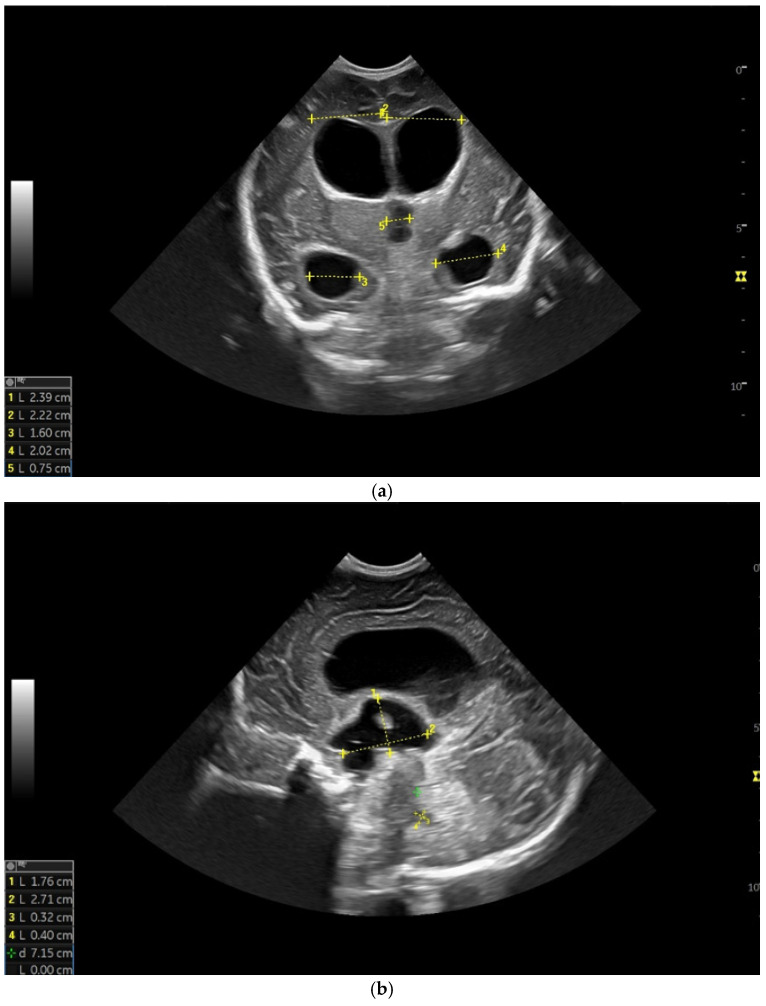

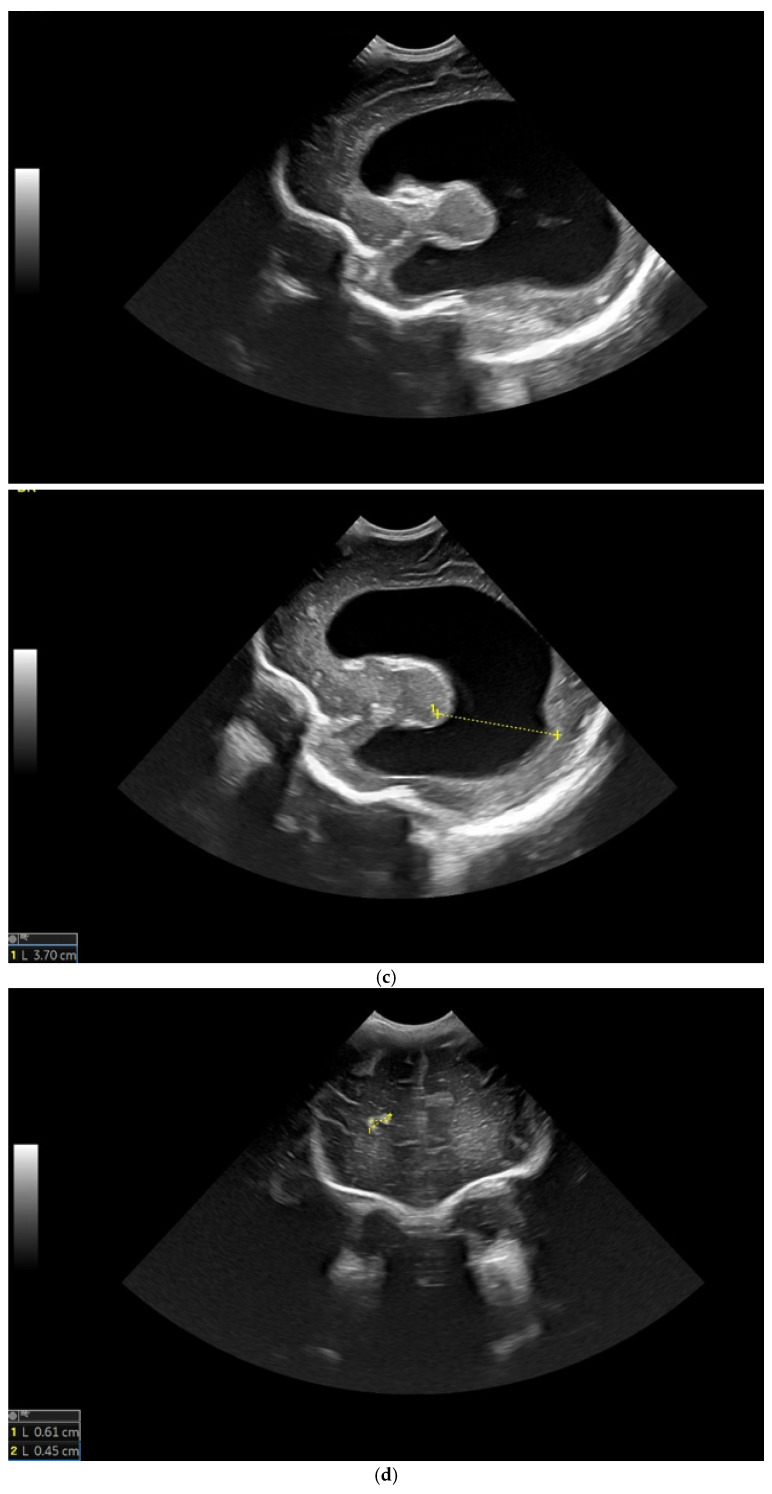

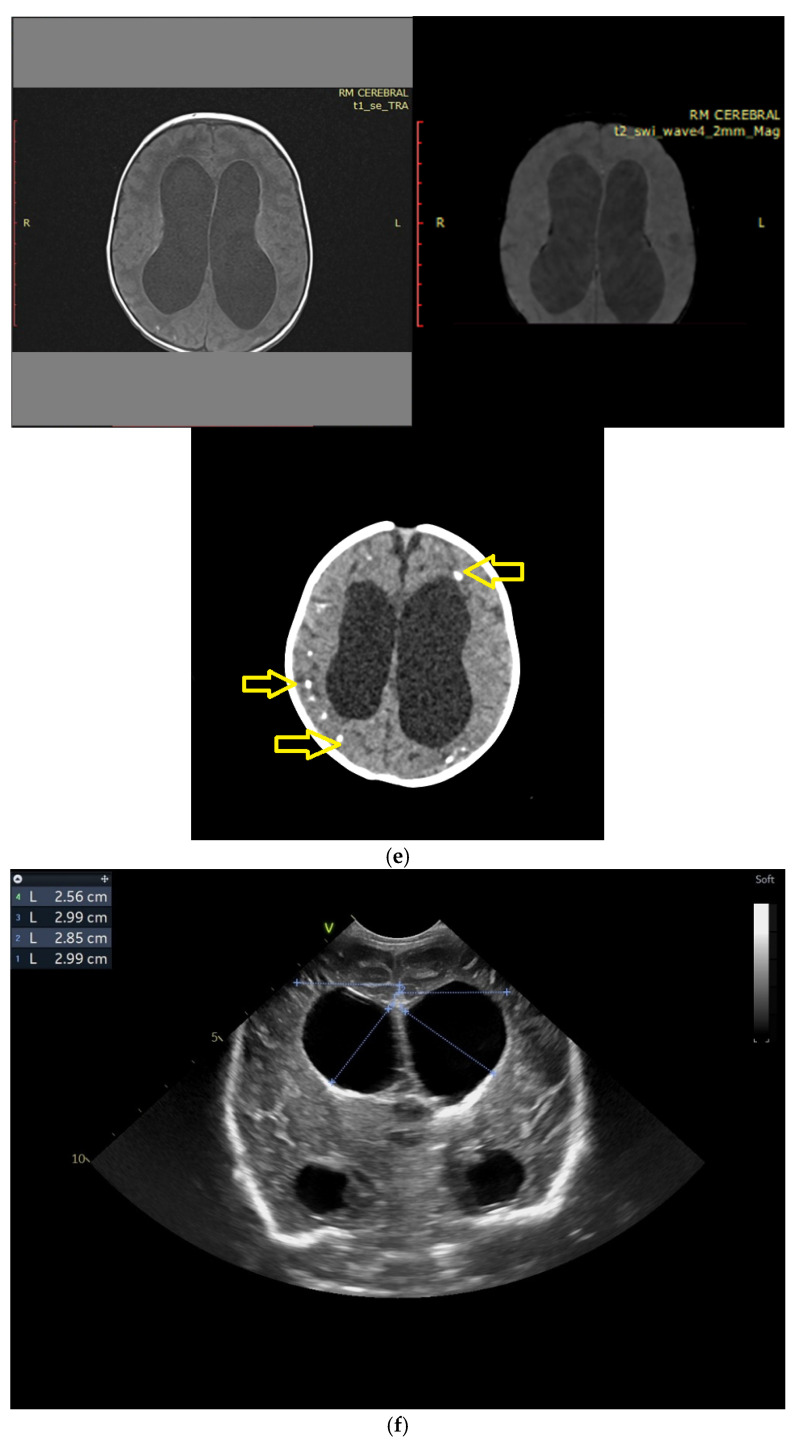

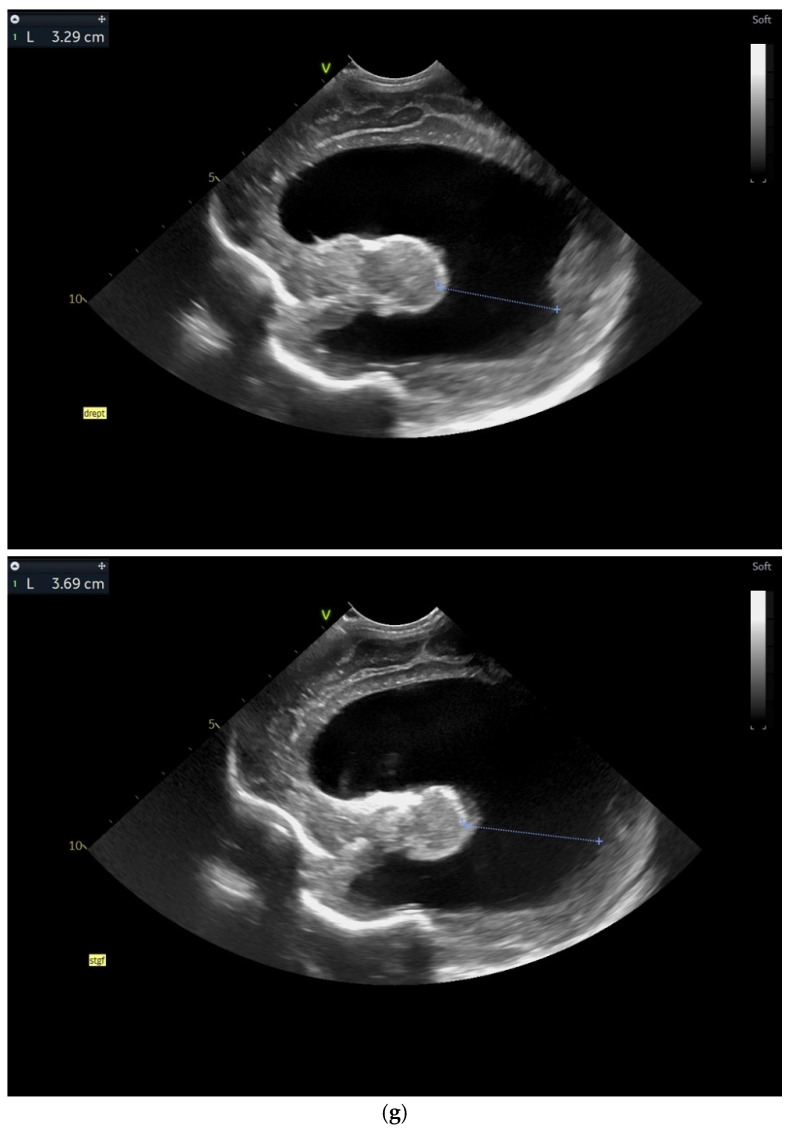
(**a**) Cranial ultrasound. Coronal view. Lateral ventricles and third ventricle enlargement. L1, L2—Levine index measurement. L3, L4—posterior horn of the lateral ventricles’ width measurement. L5—third ventricle width measurement (personal image collection). (**b**) Cranial ultrasound. Sagittal view. Enlarged third and fourth ventricles. L1, L2—third ventricle measurements. L3, L4—fourth ventricle measurements (personal image collection). (**c**) Cranial ultrasound. Sagittal view. Enlarged lateral ventricles. Up—left ventricle. Down—right ventricle (personal image collection). (**d**) Cranial ultrasound. Coronal view. Punctuate hyperechoic images were observed in the right lobe (personal image collection). (**e**). Up left—MRI T1 sequence. Up right—MRI T2 sequence—susceptibility weight imaging (SWI). Down—CT scan. Transversal view. Yellow arrows—multiple calcifications can be seen in the CT scan (personal image collection). (**f**) Cranial ultrasound. Coronal view. Lateral ventricles. L1, L2—Levine index measurement. L3, L4—anterior horn width measurement. Blue arrow—intraventricular shunt (personal image collection). (**g**) Cranial ultrasound. Sagittal view. Thalamo-occipital distance measurements. Up—right ventricle. Down—left ventricle (personal image collection).

Five weeks after birth, the patient presented for the first cranial ultrasound examination, which revealed enlarged ventricles and the presence of a hyperechoic image in the right frontal lobe, suggestive of calcifications. The parenchymal tissue was reduced (Figure 4a–d). The anterior fontanelle was wide, with large sutures. The neurological assessment showed that the infant could keep eye contact and would also hold its head during the traction-to-sit maneuver but was not able to lift its head while in the prone position. The passive tone assessment showed the “scarf sign”, with the elbow at the midline and a popliteal angle range for both legs of 90–100°. Deep tendon reflexes were present. The general movement (GM) assessment revealed a poor repertoire pattern with minimal mobility in the lower limbs. Treatment for Toxoplasma was initiated.

At six weeks after birth, an MRI scan was performed, while a CT scan was performed at 14 weeks of life, both providing valuable information regarding the sequelae of Toxoplasmosis (Figure 4e). Even though a few signs were observed on the MRI, the CT scan better outlined the presence of the parenchymal calcifications. A neurosurgical intervention was intended, a ventricle puncture was performed, and a shunt was placed in the right ventricle. The cranial ultrasound at two months of life did not show notable improvements, however (Figure 4f,g). The neurological examinations showed axial hypotonia and limb hypertonia.

### 2.5. Case 5

**Figure 5:**
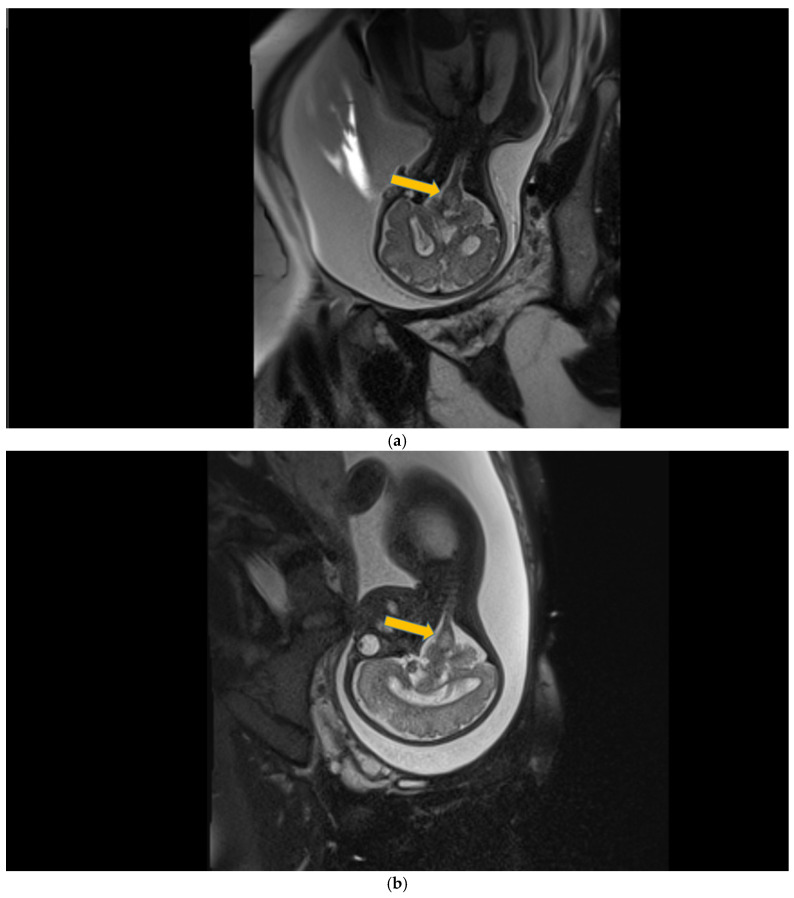

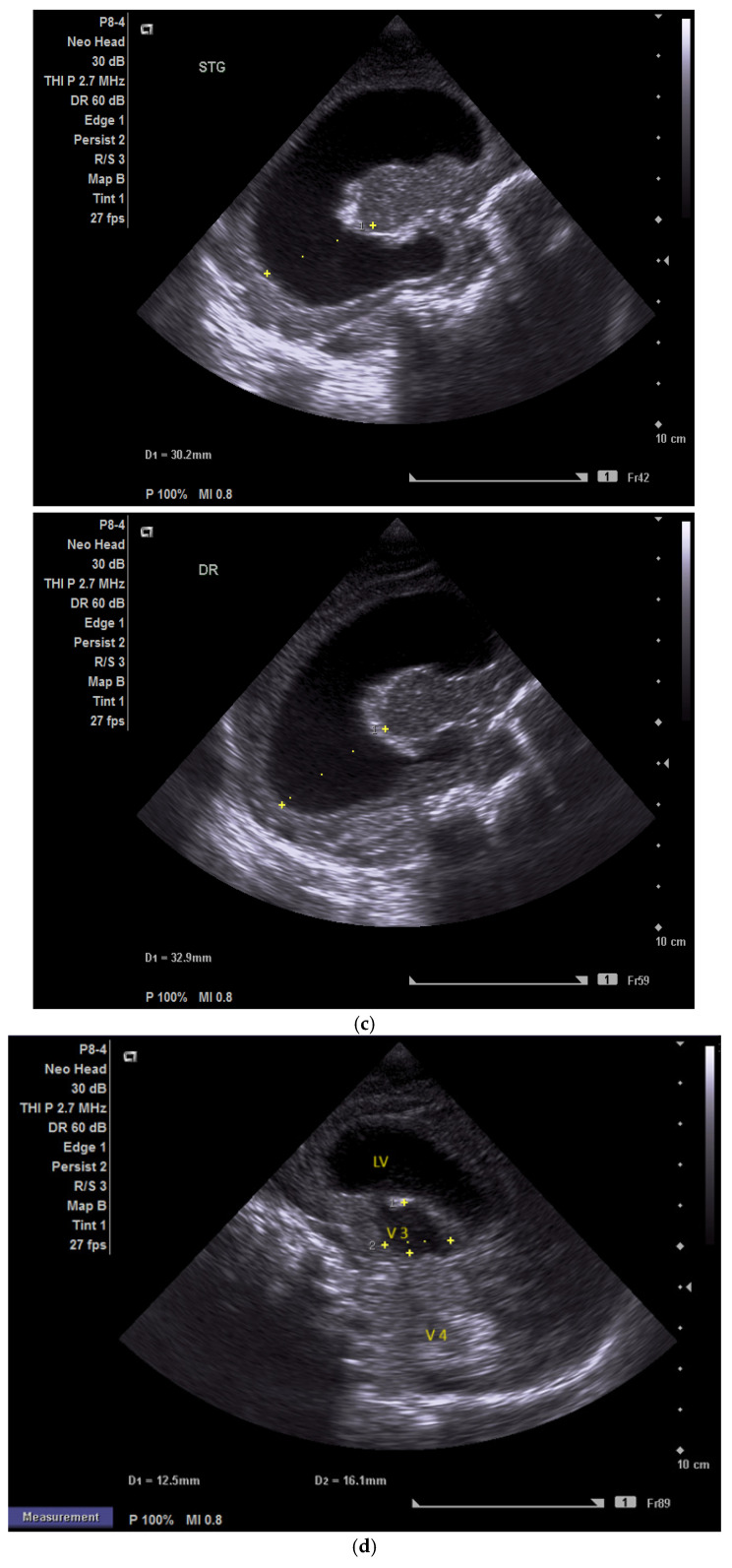

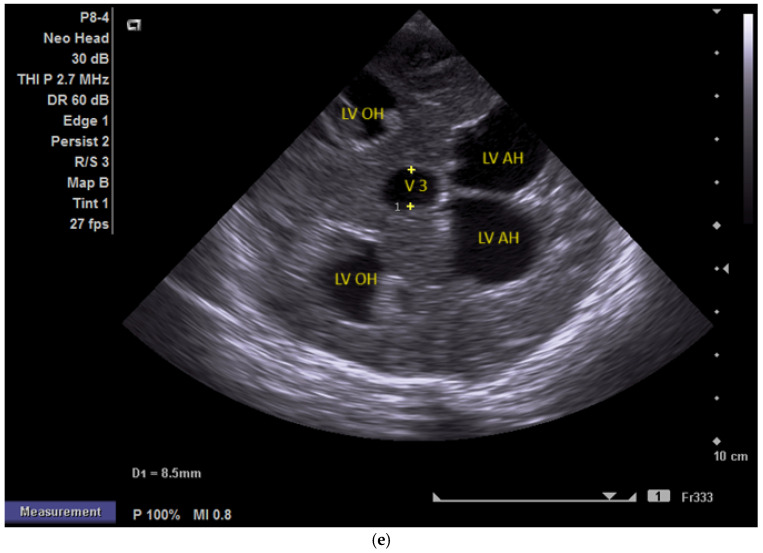
(**a**,**b**) MRI—T2 sequence. Sagittal view. Antenatal. Yellow arrows indicate the place of the suspected tumor (personal image collection). (**c**) Cranial ultrasound. Sagittal view. Up—enlarged left ventricle. D1—thamalo-occipital distance. Down—enlarged right ventricle. D1—thalamo-occipital distance (personal image collection). (**d**) Cranial ultrasound. Sagittal view. Enlarged third ventricle. Fourth ventricle is normal. D1, D2—measurements of the third ventricle (personal image collection). (**e**) Cranial ultrasound. Transtemporal view. Enlarged third ventricle. Sylvian aqueduct is not visible. D1—measurement of third ventricle. LV AH—lateral ventricle anterior horn. LV OH—lateral ventricle occipital horn. V 3—third ventricle (personal image collection).

Case 5 involved a 35-week gestational age newborn who presented with birth asphyxia and required resuscitation after delivery, which involved a balloon and mask and then a balloon and laryngeal mask, and afterwards mechanical ventilation in the NICU (Neonatal Intensive Care Unit) with SIMV/IPPV (synchronized intermittent mandatory ventilation/invasive positive pressure ventilation), who was antenatally diagnosed through MRI with subacute polyhydramnios and ventriculomegaly due to a suspected medulla oblongata tumor (Figure 5a).

After stabilization, the cranial ultrasound exam revealed enlargement of the lateral ventricles and third ventricle, with a normal fourth ventricle. There was no visualization of the Sylvian aqueduct, which raised the suspicion of aqueduct atresia or stenosis (Figure 5c–e). There was no improvement seen in the cranial ultrasound examinations that followed.

The aEEG pattern was initially a discontinuous normal voltage, which was normal for the newborn’s gestational age. At 12 h of life, there were short episodes of electrical seizures with no clinical manifestations. The seizures ceased after the administration of Phenobarbital but the pattern changed to burst suppression for approximately an hour and then returned to a discontinuous normal voltage with rudimentary sleep–wake cycles. Due to the absence of seizures, the aEEG was stopped after 48 h.

Unfortunately, the general condition of the newborn was very unstable, which was the reason why the patient was transferred to the Maria Sklodowska Curie Hospital for Children, Bucharest, for further investigations and treatment.

## 3. Discussions

Our study underscores the importance of combining multiple diagnostic methods and neurological assessments to improve the early intervention and management strategies, with the potential to enhance the outcomes for neonates with hydrocephalus. By incorporating imaging alongside clinical assessments, we offer a more robust approach to understanding the disease’s progression, which can lead to more tailored treatment protocols and better prognostic predictions along with a better correlation between ultrasound imaging and clinical or neurological assessments.

The novelty of our approach is that we considered ultrasound not merely as a radiologic tool but as a prolongation of clinical examination. By ensuring that the same physician or team performed both the ultrasound and the neurological evaluation, we enhanced the diagnostic accuracy and decision-making regarding the timing of the therapeutic intervention. This reflects the current trend in neonatal neurology, which emphasizes early intervention and close monitoring of the ventricular dilation and its clinical impact to improve the long-term prognosis.

In medical settings where there is no access to MRI, ultrasound can provide valuable information in most cases. We tried to avoid as much as possible the use of CT due to radiation exposure, although in cases such as case 4 it could bring a better view regarding the complications of the disease.

One of the strengths of our case series is the close follow-up of all patients, thereby offering more comprehensive monitoring throughout their clinical evolution. Moreover, the ultrasound imaging and neurological examination performed during the follow-ups brought to our attention a better correlation between the imaging findings and clinical progression. However, like many studies, our research has certain drawbacks. One such drawback is the gestational age range due to the fact that the minimum gestational age in our case series was 30 weeks, thereby restricting our ability to generalize the findings to preterm infants below this threshold, who frequently present with different clinical trajectories and outcomes. Additionally, the absence of MRI—due to limited resources—in the first three cases limited our ability to assess subtle parenchymal injuries that could provide further insights into neurodevelopmental prognoses.

Concerning the therapeutic approach, a one-size-fits-all strategy is not feasible because of the heterogeneity of etiologies and individual disease progression. In the case of post-hemorrhage dilation, we performed lumbar punctures, being guided by the RI and ultrasound imaging. However, it is crucial to determine whether the hemorrhage appeared before or after birth due to the impact it can have on the evolution of the disease, as well as the neurodevelopment of the patient. In the case of prenatal ICH (intracranial hemorrhage), the risk of mortality and neurodevelopmental outcome anomalies are increased [29].

Another key issue is represented by finding the aetiology to guide the optimal therapeutic approach. In some cases, a multimodal approach was necessary; case 4, for instance, required a combination of medical treatment, ventricular puncture, and a shunting device. Regarding the prognosis of hydrocephalus, it is closely tied to the aetiology [2]. In the case of congenital hydrocephalus, genetic testing and counselling are important. Advanced genetic tools such as the “hydrocephalus panel” bring valuable information for the diagnosis, treatment, and evolution courses [2,30]. Among the inherited forms, X-linked hydrocephalus associated with the stenosis of the aqueduct is the most common [6]. The progress of genetic research and testing brings along new opportunities for targeted pharmacological treatment. It is, however, important to underscore the fact that the burden of congenital hydrocephalus is higher in low- or middle-income countries due to the lesser resources for genetic testing [11]. Furthermore, genetically influenced abnormalities in neurogenesis are associated with poorer neurodevelopmental outcomes in congenital hydrocephalus cases [10,31,32,33,34]. The KANET (Kurjak Antenatal Neurodevelopmental Test) and Prechtl’s Assessment of General Movements are valuable tools for monitoring fetal and neonatal neurological function, with the first being used in the antenatal period using a 4D ultrasound examination to observe the fetal movements, while the latter is based upon clinical observations of the newborns and infants’ movements [35,36,37]. The KANET proves its utility by offering prenatal information about the neurological status of the fetus, and the types of movement the fetus presents during its evolution, giving the opportunity for in utero or early post-partum intervention [35]. Prechtl’s Assessment of General Movements focuses on the post-natal spontaneous movements. If performed correctly, it enables early inclusion in rehabilitation programs, potentially improving the long-term neurological outcomes [37,38,39,40]. Currently, there is increasing sustainment for using neonatal point-of-care ultrasound (POCUS) due to its utility and feasibility, as well as its reasonable sensitivity and reliability in detecting several diseases [40,41]. A key aspect emphasized in this study is the comparison between the ultrasound findings and other imaging modalities, such as MRI and CT. Regarding the case of the newborn with Toxoplasmosis, for instance, the ultrasound raised important diagnostic suspicions, prompting further targeted investigations.

We also consider that encouraging neonatal resident doctors to perform cranial ultrasounds under supervision would bring better results in the future, especially in medical settings with low resources, providing better management of the cases and an early intervention where it is needed.

Despite the limited cohort size, these findings highlight the necessity of larger analytical studies to systematically assess the role of ultrasound in comparison to gold-standard imaging techniques for neonatal evaluation and follow-up. Future research in this direction could help refine protocols for the optimized use of ultrasound in clinical practice.

## 4. Conclusions

Cranial ultrasound remains one of the most powerful and accessible tools for the diagnosis and monitoring of hydrocephalus, offering real-time insights into ventricular dynamics, and in some cases aiding in the identification of underlying etiologies. Its ease of use, bedside availability, and ability to facilitate close follow-up make it an essential component of neonatal and pediatric neurology. However, ultrasound alone may not always provide a comprehensive assessment of hydrocephalus etiology, particularly in cases requiring detailed evaluations of parenchymal injuries, cerebral hypoxia, or complex structural anomalies. In such situations, MRI or CT imaging remains crucial for confirming the diagnosis and guiding management decisions.

Our case series highlights the heterogeneous nature of hydrocephalus, emphasizing that the disease’s evolution is closely linked to its underlying pathophysiological mechanisms. Given this variability, a case-specific approach is essential for optimizing patient outcomes. A thorough evaluation of the patient’s medical history, combined with appropriate neuroimaging and genetic testing when available, is recommended to ensure a precise diagnosis, timely intervention, and improved prognostic assessment.

## Data Availability

The original contributions presented in the study are included in the article, further inquiries can be directed to the corresponding author.
